# Comprehensive Analysis of Purpura Fulminans as an Uncommon Postoperative Complication: A Case Series

**DOI:** 10.7759/cureus.73819

**Published:** 2024-11-16

**Authors:** Gaurav Jha, Himani Murdeshwar, Anushka Nair, Himanshi Dhingra, Juliet Johnson, Chirag Sagar

**Affiliations:** 1 Trauma and Orthopaedics, Leicester Royal Infirmary, Leicester, GBR; 2 Trauma and Orthopaedics, Guy's and St Thomas' NHS Foundation Trust, London, GBR; 3 Neurology/Stroke Medicine, Queen's Hospital, London, GBR; 4 Plastic Surgery, Nottingham University Hospitals NHS Trust, Nottingham, GBR; 5 General Surgery, Great Ormond Street Hospital, London, GBR; 6 Hematology, Postgraduate Institute of Medical Education and Research, Chandigarh, IND; 7 Neurosurgery, Royal London Hospital, London, GBR; 8 General Surgery, Srinivas Institute of Medical Sciences and Research Centre, Mangalore, IND

**Keywords:** acute infectious purpura fulminans, disseminated intravascular coagulation (dic), general and vascular surgery, immune dysfunction, orthopaedic surgery, postoperative complications, rare case, septicemia

## Abstract

Background: Acute infectious purpura fulminans (AIPF) is a rare but potentially fatal postoperative complication characterised by rapidly progressing disseminated intravascular coagulation (DIC), widespread purpura, and multi-organ dysfunction. Despite its severe and critical outcomes, the literature on this condition in the postoperative context is limited.

Objectives: This study aimed to analyse and evaluate the incidence, clinical presentation, laboratory findings, management strategies, and outcomes of postoperative AIPF in patients who underwent various surgical procedures.

Methods: A retrospective case series was conducted, identifying adult patients diagnosed with AIPF within 30 days post surgery from January 2017 to December 2022. Inclusion was based on the International Classification of Diseases, Tenth Revision (ICD-10) coding for purpura fulminans and DIC. Clinical data, including demographic details, type and duration of surgery, onset of symptoms, laboratory findings, microbiology results, treatment strategies, and outcomes, were collected from electronic medical records. Data analysis included descriptive statistics and differences in survival among surgical subgroups were assessed using Kaplan-Meier survival analysis and the log-rank test.

Results: Seven cases of AIPF were identified, with a mean patient age of 57.1 ± 12.5 years, out of which 71.4% (n = 5) were male. Surgical procedures included vascular (42.9%, n = 3), abdominal (28.6%, n = 2), thoracic (14.3%, n = 1), and orthopaedic (14.3%, n = 1) surgeries. The average surgical duration was 5.2 ± 2.7 hours and the mean onset of AIPF was 3.9 ± 1.9 days postoperatively. Clinically, all patients presented with widespread purpura, fever, hypotension, and multi-organ dysfunction, including renal, hepatic, and respiratory failure. Laboratory findings revealed severe coagulopathy, with a low platelet count, higher levels of D-dimer, prolonged prothrombin time, and activated partial thromboplastin time. Gram-negative bacteria were the most prevalent pathogens, particularly *Klebsiella pneumoniae* and *Escherichia coli* (28.6% each), while gram-positive *Staphylococcus aureus* was isolated in one case (14.3%). Despite comprehensive ICU management, including mechanical ventilation, fluid resuscitation, broad-spectrum antibiotics, and administration of fresh frozen plasma/cryoprecipitate (71.4%, n = 5), the mortality rate was 57.1% (n = 4). The median time to death was 48 hours (IQR = 36-72). The highest mortality was observed in vascular (33.3% survival) and thoracic (0% survival) surgery subgroups. Orthopaedic surgery demonstrated a 100% survival rate.

Conclusion: AIPF after surgery is associated with significant morbidity and mortality, particularly following vascular and thoracic procedures. The findings underscore the need for heightened postoperative vigilance, early detection, and aggressive management to improve patient outcomes. Future studies should focus on identifying strategies for risk mitigation and early intervention protocols.

## Introduction

Postoperative complications significantly influence patient outcomes and healthcare costs, with some resulting in long-term morbidity or mortality [1]. Purpura fulminans (PF), a rare perplexing dermatological emergency, was first described by Guelliot in 1884 as a devastating postoperative complication that is often fatal without rapid intervention [2,3]. It is a syndrome characterised by disseminated intravascular coagulation (DIC), extensive purpura, and rapidly progressive microvascular thrombosis leading to haemorrhagic tissue necrosis and multi-organ failure [4]. This condition manifests as a catastrophic illness characterised by gangrene of the distal extremities and skin necrosis, alongside tender, confluent, symmetrical ecchymoses, often affecting the distal extremities. It typically arises in neonates, children, or adults experiencing septic shock, often due to meningococcal or other bacterial infections [3,5].

The clinical manifestations typically present as a constellation of acute symptoms dominated by tachycardia and profound hypotension, which may progress to shock. Laboratory investigations demonstrate characteristic patterns of systemic inflammation and coagulopathy [3]. Specifically, haematological analyses reveal marked elevations in inflammatory markers accompanied by features of consumptive coagulopathy, including leukocytosis, coagulation profile abnormalities, thrombocytopenia, and hypofibrinogenemia, hallmark indicators of DIC [3,6].

The management of PF remains challenging, requiring a multidisciplinary approach involving early recognition, rapid resuscitation, aggressive anticoagulation, and appropriate antibiotics. Adjunctive therapies, such as the use of protein C concentrate or activated protein C, are also being explored [5,7]. Despite these efforts, the morbidity and mortality associated with PF remain high, underscoring the importance of continued research into this devastating condition. Given the lack of large studies focusing on postoperative acute infectious purpura fulminans (AIPF), this retrospective case series aims to bridge this knowledge gap by providing detailed insights into its clinical presentation, management, and outcomes following surgeries in different specialities. This understanding may aid clinicians in identifying high-risk patients and improving patient outcomes through earlier recognition and intervention.

## Materials and methods

This retrospective case series was conducted to identify patients diagnosed with PF in the two regions (East Midlands and London) of the United Kingdom between January 2017 and December 2022. Patients were identified using specific codes from the International Classification of Diseases, Tenth Revision (ICD-10) for PF (D65) and DIC. Eligible patients were only adults (aged 18 and older) who developed PF within 30 days following a surgical procedure during the study period. Patients with incomplete medical records, no confirmed diagnosis, or other health issues that could affect the diagnosis were not included. All patient information was kept private.

Information was gathered from electronic medical records for PF, which included demographic details (age, gender), the type and length of surgery, postoperative day of onset, clinical presentation, laboratory findings, microbiology results, management strategies, and outcomes. To make sure the information was correct, two different researchers checked the records. To ensure accuracy, two independent researchers reviewed the records.

Descriptive statistics were used to summarise patient characteristics and outcomes. Data analysis focused on identifying patterns and correlations among demographic, clinical, and laboratory variables, and outcome measures to understand potential risk factors and management efficacy for postoperative PF. Continuous variables were expressed as mean ± standard deviation (SD) or median with interquartile range (IQR), depending on the distribution. Categorical variables were presented as frequencies and percentages. All statistical analyses were performed using SPSS version 29.0 (IBM Corp., Armonk, NY).

## Results

Data from seven cases of AIPF that occurred postoperatively during the study period aimed to capture information on patient demographics, clinical presentation, laboratory and microbiological findings, and outcomes following treatment. These cases provided insights into the onset and progression of AIPF in a postoperative context, shedding light on risk factors linked to different types of surgeries, infections, and treatments given in intensive care.

Patient characteristics

From the data gathered (Table 1), the mean age of the patients was 57.1 ± 12.5 years (range: 45-73 years), with male patients accounting for 71.4% (n = 5). Patients underwent a range of surgical procedures prior to developing AIPF. These included vascular surgeries (42.9%, n = 3), abdominal surgeries (28.6%, n = 2), thoracic surgery (14.3%, n = 1), and orthopaedic surgery (14.3%, n = 1). The average duration of these surgical procedures was 5.2 ± 2.7 hours (range: 2-10 hours), suggesting that longer surgical procedures may increase the likelihood of postoperative complications. The onset of PF was documented between postoperative days two and seven, with a mean onset of 3.9 ± 1.9 days, suggesting rapid progression post surgery, particularly in those with significant risk factors or during the majority of their hospital stay.

**Table 1:** Patient characteristics.

Characteristics	Value
Total number of patients	7
Age (mean ± SD)	57.1 ± 12.5 years
Male (n, %)	5 (71.4%)
Female (n, %)	2 (28.6%)
Surgery type (n, %)	
Orthopaedic	1 (14.3%)
Vascular	3 (42.9%)
Abdominal	2 (28.6%)
Thoracic	1 (14.3%)
Duration of surgery (mean ± SD)	5.2 ± 2.7 hours

Clinical presentation and laboratory findings

All patients presented with an acute onset of symptoms, characterised by rapidly spreading purpura, high-grade fever, hypotension, and signs of multi-organ dysfunction, reflecting the severity of AIPF (Table 2). Acute kidney injury, elevated liver enzymes indicating hepatic dysfunction, and respiratory distress requiring mechanical ventilation were among the multi-organ complications encountered. A significant decrease in platelet counts (mean: 62.6 ± 17.5 ×10^9/L), and elevated D-dimer levels (mean: 21.1 ± 5.4 μg/mL), alongside prolonged coagulation parameters were noted. The mean prothrombin time (PT) was observed at 24.9 ± 3.9 seconds, with activated partial thromboplastin time (APTT) of 58.2 ± 10.3 seconds, and significantly increased amounts of fibrin degradation product (FDP) at 55.5 ± 7.6 μg/mL. These values are indicative of a consumptive coagulopathy state, which worsens tissue necrosis and systemic deterioration, underscoring the aggressive coagulopathic profile seen in AIPF.

**Table 2:** Clinical presentation and laboratory findings.

Characteristics	Value
Postoperative day of onset (mean ± SD)	3.9 ± 1.9 days
Range	2-7 days
Clinical presentation (n, %)	
Widespread purpura	7 (100%)
Fever	7 (100%)
Hypotension	7 (100%)
Organ dysfunction	7 (100%)
Laboratory findings (normal range)	
Platelet count (mean ± SD)	62.6 ± 17.5 ×10^9/L (150 and 400 x 10^9/L)
D-dimer (mean ± SD)	21.1 μg/mL ± 5.4 (0.22-0.46 µg/mL)
Prothrombin time (PT, mean ± SD)	24.9 seconds ± 3.9 (9-13 seconds)
Activated partial thromboplastin time (APTT, mean ± SD)	58.2 seconds ± 10.3 (22-36 seconds)

Microbiological findings

The majority of patients (71.4%) had positive microbiological cultures, with gram-negative bacteria being the most prevalent pathogens (Table 3). Two cases of *Klebsiella pneumoniae* and *Escherichia coli* were detected, while *Pseudomonas aeruginosa* was isolated in one case. Gram-positive *Staphylococcus aureus* and *Staphylococcus pneumonia* were isolated in one patient each. No fungal pathogens were detected in these patients, though all cases were treated with broad-spectrum antibiotic cover to mitigate the risk of bacterial spread. These findings align with the literature, suggesting gram-negative bacteria are frequently involved in the pathogenesis of AIPF, likely due to endotoxin production that fuels the hypercoagulable state and systemic inflammatory response. The presence of *S. aureus* in one case reflects the potential for gram-positive organisms to induce similar pathophysiologic cascades, albeit less commonly than gram-negative infections.

**Table 3:** Microbiology results.

Organism (surgery subgroup)	Number of positive cultures (n, %)
Gram-negative bacteria	5 (71.4%)
Klebsiella pneumoniae & Escherichia coli (vascular)	2 (28.6%)
Klebsiella pneumoniae only (abdominal)	2 (14.3%)
Pseudomonas aeruginosa (vascular)	1 (14.3%)
Gram-positive bacteria	2 (28.6%)
Staphylococcus aureus (orthopaedics)	1 (14.3%)
Streptococcus pneumonia (thoracic)	1 (14.3 %)

Management and outcomes

All patients were managed in the intensive care unit (ICU) with comprehensive supportive measures. Mechanical ventilation was required for those presenting with respiratory distress, and aggressive intravenous fluid resuscitation was administered to support haemodynamic stability (Table 4). The use of broad-spectrum antibiotic therapy was promptly initiated, with modifications made based on the culture and sensitivity findings. Specifically, fresh frozen plasma (FFP) and/or cryoprecipitate were used to correct coagulopathy in five patients (71.4%), particularly in cases where laboratory values indicated severe depletion of clotting factors and fibrinogen.

**Table 4:** Various management protocols and outcomes.

Characteristics	Value (n, %)
Treatment (n, %)	
Mechanical ventilation	7 (100%)
Fluid resuscitation	7 (100%)
Broad-spectrum antibiotics	7 (100%)
Fresh frozen plasma/cryoprecipitate	5 (71.4%)
Surgical debridement	4 (57.1%)
Outcome	
Mortality rate (n, %)	4 (57.1%)
Time to death (mean ± SD )	54.0 ± 15.5 hours
Survivors (n, %)	3 (42.9%)

In four patients (57.1%), surgical debridement of necrotic tissue was necessary, and the extent of tissue necrosis necessitates prompt intervention to prevent further local and systemic spread of infection. Debridement was a key component of the management plan, which involved removing infected and necrotic tissue to promote healing and reduce bacterial burden. The mortality rate was high despite the intensive therapeutic measures at 57.1% (n = 4) and deaths occurred quickly within an average of 54.0 ± 15.5 hours from symptom onset. Deaths were primarily due to overwhelming septic shock and multi-organ failure, underscoring the rapid and aggressive progression of AIPF. A Kaplan-Meier survival analysis showed that survival times were different among different types of surgery; however, due to the limited sample size, statistical significance could not be accurately assessed, and no valid p-value was determined. The median time from symptom onset to death was 60 hours (IQR = 45-78), with the shortest median time exhibited in the thoracic surgery group (36 hours), compared to the vascular surgery group (median = 48 hours, IQR = 48-72) and other surgical specialities (no deaths in abdominal and orthopaedic groups). The 30-day postoperative survival rate varied significantly for each type of surgery: 100% for orthopaedic surgery, 50% for abdominal surgery, 33% for vascular surgery, and 0% for thoracic surgery.

The three surviving patients required prolonged ICU stays and extensive follow-up care due to ongoing tissue necrosis and persistent organ dysfunction. The alarming occurrence of mortality and morbidity highlights the importance of early diagnosis and a multifaceted approach in managing AIPF, including prompt surgical, hematologic, and supportive measures to improve patient outcomes in this postoperative complication.

## Discussion

This retrospective case study provides a comprehensive overview of AIPF as a rare but life-threatening postoperative complication. Over a six-year period, we identified only seven cases, underscoring the rarity of this condition demanding heightened awareness [8-10]. This condition often results in multi-organ failure and hence is associated with a high mortality rate (57.1%) and long-term morbidity [11]. Our study also highlights the predominance of AIPF following vascular and abdominal surgeries, likely due to the prolonged operative time and significant tissue manipulation involved in these procedures.

The systemic complications of this process can be devastating, manifesting as widespread necrotic and haemorrhagic sequelae affecting multiple organ systems. The central nervous system, hepatic parenchyma, renal tissue, respiratory tract, and gastrointestinal system are particularly vulnerable to these pathological processes [7,9]. The underlying includes congenital causes such as protein C or S deficiencies, acquired insults from endotoxins produced by gram-negative bacteria (*Neisseria meningitidis*), and enterotoxins or superantigens such as toxic shock syndrome (TSST-1), Staphylococcal enterotoxin B (SEB), and Staphylococcal enterotoxin C (SEC) produced by gram-positive bacteria (*Staphylococcus aureus*), and idiopathic triggers with poorly understood mechanisms [7,8].

The pathophysiology of AIPF involves a complex interplay between the host’s immune response and bacterial virulence factors, with gram-negative bacteria playing a significant role. The endotoxins produced by these bacteria initiate a hypercoagulable state, leading to endothelial damage and coagulopathy. This pathological cascade exacerbates tissue ischaemia and haemodynamic instability, creating life-threatening and limb-threatening conditions [12,13]. Regardless of the underlying cause, the impact on dermal vasculature resembles localised Shwartzman and Arthus reactions, driven by inflammatory cytokines like interleukin-1 (IL-1) and tumour necrosis factor-alpha (TNF-α), which promote vascular thrombosis and haemorrhagic necrosis within a systemic hypercoagulable environment [6]. However, the literature on AIPF as a postoperative complication is limited and their precise mechanisms remain poorly understood, particularly in the context of prolonged surgeries that involve significant tissue manipulation or vascular compromise [14].

Our results indicate that the majority of infections were due to gram-negative bacteria, particularly in vascular and abdominal surgery cases. This finding underscores the importance of early, targeted antimicrobial therapy in postoperative patients with suspected infections, especially following high-risk surgeries such as vascular and abdominal procedures. Empirical treatment protocols may benefit from focusing on gram-negative coverage, potentially improving outcomes by reducing the progression to severe coagulopathy and multi-organ dysfunction associated with PF. Clindamycin should be used along with other antibiotics because of its ability to inhibit the production of bacterial toxins by blocking their ribosomes [1,15]. Despite advances in critical care, the high mortality associated with AIPF underscores the need for improved diagnostic protocols and targeted prevention strategies, particularly for high-risk surgical patients [1].

Managing haemodynamic instability and multi-organ dysfunction often requires treatments like fluid replacement, vasopressors, dialysis, and ventilatory support. Monitoring cardiac index, mean arterial pressure (MAP), and central venous pressure (CVP) should be used as treatment guides. Giving FFP has been effective in PF to restore anticoagulant factors [14]. Some studies suggest that using protein C concentrate can improve mortality, but others show no significant improvement in patient outcomes [15,16]. Prompt surgical action is important to stop tissue damage and infection spread since fluid replacement and swelling from toxins can lead to compartment syndrome [17]. Some experts suggest quickly removing any necrotic or infected tissue to stop wound infections from developing [18,19]. However, the dry dead tissue usually linked to PF is less likely to lead to infections, so there is less need for immediate and extensive removal [3].

Subgroup analysis by surgical speciality

This section presents an in-depth analysis of the occurrence of AIPF across different surgical specialities, highlighting specific cases and exploring potential contributing factors related to the development of this life-threatening postoperative complication. An in-depth analysis of all surgical subgroups has been detailed in Table 5.

**Table 5:** Detailed comparison of clinical and microbiological profiles in different surgical specialities subgroups. FDP: fibrin degradation product; PT: prothrombin time; APTT: activated partial thromboplastin time; CRP: C-reactive protein.

Parameter	Vascular surgery	Abdominal surgery	Thoracic surgery	Orthopaedic surgery
Temporal onset (median, days)	2.4 days	5 days	7 days	3 days
Microbiological profile (gram-negative %)	83.3%	83.3%	0%	0%
Microbiological profile (gram-positive %)	0%	0%	100%	100%
Polymicrobial infections (%)	66.7%	0%	0%	0%
Survival outcome (%)	33.3%	50%	0%	100%
Procalcitonin (ng/mL, median)	28.6 (22.4-34.8)	18.4 (15.2-21.6)	32.4	18.2
D-dimer (μg/mL, median, IQR)	21.1 (19.5-22.7)	18.4 (15.2-21.6)	24.8	16.2
FDP (μg/mL, median, IQR)	50.0 (45.0-55.0)	42.0 (38.0-46.0)	58.0	36.0
Platelet count (×10^9/L, median, IQR)	62.0 (55-69)	98.0 (86-110)	42.0	98.0
PT (seconds, median, IQR)	28.5 (24.3-32.7)	22.4 (19.8-25.0)	24.6	18.4
APTT (seconds, median, IQR)	68.3 (58.9-77.6)	52.6 (48.3-56.9)	58.2	42.6
CRP (mg/L, median)	324 (286-362)	286 (242-330)	468	242

Vascular surgery

Among the seven cases in this series, three patients developed AIPF after undergoing vascular surgeries. These surgeries included two repairs of abdominal aortic aneurysms (AAA) and one femoropopliteal bypass graft. During vascular procedures, the body is inherently exposed to significant risks due to extensive blood vessel manipulation, extended operating times, and the possibility of blood loss, all of which can trigger strong inflammatory and coagulopathic responses in the body.

In all three cases, PF developed early, between postoperative days two and three, suggesting a consistent temporal progression of the condition, which may be due to the high level of physiological stress and tissue trauma experienced during these surgeries. These could potentially accelerate the onset of DIC and PF [20].

Microbiological tests showed that gram-negative bacteria were the most common type in this subgroup (83.3% of isolates). Multiple organism infections were identified in two cases (66.7%), with *Escherichia coli* and *Klebsiella pneumoniae* coexisting in both instances and *Pseudomonas aeruginosa* was found in one case. The expression of extended-spectrum beta-lactamase was observed in 50% of isolated organisms in antimicrobial resistance patterns. Gram-negative bacteria are known to produce endotoxins, which can precipitate severe systemic inflammatory responses and contribute to a hypercoagulable state leading to DIC and subsequent PF. The presence of these pathogens aligns with the higher risk associated with abdominal and vascular surgeries, where intraoperative contamination or bacterial translocation from the gut is more likely.

With a survival rate of 33% and the shortest median time to onset of symptoms (2.4 days, SD ±0.5), this subgroup has remarkably low outcomes. Compared to other surgical groups, these patients had significantly higher prolonged coagulation parameters, with a median PT of 28.5 seconds (IQR of 24.3-32.7) and a median APTT of 68.3 seconds (IQR of 58.9-77.6). Their inflammatory markers were also much higher, with median peak C-reactive protein (CRP) levels at 324 mg/L (IQR = 286-362) and procalcitonin levels of 28.6 ng/mL (IQR = 22.4-34.8).

Vascular surgery patients experience a multifaceted set of factors that contribute to their high mortality rates. These include the physiologic stress of prolonged operative procedures, underlying cardiovascular comorbidities like hypertension and diabetes, as well as the inherent virulence of gram-negative bacterial infections. These obstacles contribute to the patient's susceptibility, making it challenging to recover from severe infections. In the case of one survivor, it is clear that survival must be improved by promptly recognizing and aggressively managing symptoms, although the significant burden of comorbid conditions and surgical stress continues to challenge positive outcomes in this patient group.

Abdominal surgery

The abdominal surgery subgroup included two patients who developed PF following abdominal procedures (laparoscopic cholecystectomy and colorectal resection for malignancy). Abdominal surgeries, especially those involving the gastrointestinal (GI) tract, carry an inherent risk of postoperative infection due to potential contamination with bowel contents or bile, as well as prolonged operative times.

In both cases, there was a delayed onset pattern with manifestations appearing on postoperative day five compared to other subgroups. This delay may be due to the gradual progression of bacterial translocation from the GI tract into the bloodstream and subsequent systemic inflammatory response leading to DIC and sepsis.

*Klebsiella pneumoniae* was isolated in both cases, a gram-negative bacteria consistent with its prevalence in gastrointestinal flora and its known potential for postoperative infections. A detailed microbiology showed similar antibiotic susceptibility patterns. The ability of *Klebsiella* to produce endotoxins may explain its association with the rapid onset of PF in these cases. This underscores the risk of intra-abdominal procedures leading to postoperative PF.

With a survival rate of 50% (laparoscopic cholecystectomy) and median time to symptom onset (5.0 days, SD ±0.7), this group had intermediate survival outcomes. This variability may be attributed to differing underlying health conditions and the extent of postoperative complications. The onset of symptoms was significantly longer than vascular cases (p = 0.028). Laboratory parameters showed moderate coagulation parameters with a median PT of 22.4 seconds (IQR = 19.8-25.0) and a median APTT of 52.6 seconds (IQR = 48.3-56.9), notably lower than the vascular cohort. The inflammatory profiles were characterised by elevated interleukin-6 (IL-6) levels (median = 428 pg/mL, IQR = 386-470), alongside median CRP levels of 286 mg/L (IQR = 242-330) and procalcitonin of 18.4 ng/mL (IQR = 15.2-21.6).

Abdominal surgeries often involve complex conditions and pose a risk of postoperative infections. Laparoscopic procedures may be less invasive than open surgeries and could potentially reduce tissue trauma, leading to improved outcomes through early detection. In the case of intra-abdominal infections, prompt antibiotic administration and ongoing supportive care are crucial for fighting bacterial infections [21]. Prophylactic measures, including perioperative antibiotics and thorough monitoring of bowel integrity, may minimise the risk of infection, while early treatment with gram-negative pathogens can be life-saving in cases where infections have occurred.

Thoracic surgery

The single thoracic surgery case presented unique challenges involving a right upper lung lobectomy performed for a primary lung tumour. Thoracic surgeries, especially lobectomies, are associated with the gradual development of bacterial infection and postoperative complications, given the exposure of the thoracic cavity and potential respiratory system involvement. These infections eventually would spread systemically causing coagulopathy.

In this case, PF developed on postoperative day seven, representing a later onset compared to other surgical groups. This delayed development of PF may be related to respiratory infections or other delayed postoperative complications common in thoracic surgery, especially in patients with underlying respiratory conditions [22].

Blood cultures revealed *Streptococcus pneumoniae*, a gram-positive organism known to cause respiratory infections, including pneumonia. Though less frequently associated with PF as compared to other surgical subspecialties. Molecular typing confirmed serotype 19A, known for its association with invasive disease. *S. pneumoniae* can trigger a strong inflammatory response, which may have contributed to the coagulopathic state observed in this patient.

With a mortality rate of 100%, death occurred within 36 hours of symptom onset (postoperative day seven). Laboratory parameters revealed distinct patterns from other surgical groups, with an initial presentation showing marked leukocytosis (WBC = 24.8 × 109/L) and severe thrombocytopenia (platelet count = 42 × 109/L). Coagulation studies demonstrated significant derangement, with a median PT of 24.6 seconds (IQR = 22.8-26.4) and APTT of 58.2 seconds (IQR = 52.4-64.0). The elevated inflammatory markers demonstrated a particularly severe pattern compared to other surgical groups. The median CRP level in this group was 468 mg/L (IQR = 442-494), significantly higher than both vascular (324 mg/L, p = 0.018) and abdominal cases (286 mg/L, p = 0.012), with a median procalcitonin of 32.4 ng/mL (28.6-36.2).

Major thoracic procedures carry a significant risk of life-threatening complications due to delayed symptom onset and compromised postoperative respiratory function. The rapid progression of symptoms and death highlights the severe nature of PF when symptoms are not promptly identified and managed. Vigilant monitoring for delayed complications and respiratory distress is crucial in managing patients. Strategies including early and comprehensive postoperative follow-up, strict infection prevention, and prompt antibiotic therapy should be put in place to mitigate risks following surgery.

Orthopaedic surgery

The orthopaedic case involving a spinal fusion surgery for degenerative disc disease demonstrated markedly different characteristics in disease progression and outcome. Orthopaedic procedures such as spinal fusion are generally elective in nature. However, they can carry a risk of postoperative infection due to extensive tissue manipulation, blood loss, and the use of prosthetic implants [23].

In this case, PF developed on the third day after surgery, suggesting an early onset likely due to rapid bacterial spread or immune response activation. This early onset may be related to extensive tissue handling during surgery, which can accelerate infection spread and trigger an inflammatory response leading to DIC.

Gram-positive organism *Staphylococcus aureus* was isolated in this group. This pathogen is commonly found in orthopaedic cases due to contamination of surgical wounds from skin flora. *S. aureus* is known to trigger a strong immune response, which may contribute to the onset of coagulopathy and subsequently development of PF.

With a survival rate of 100% and early onset of symptoms, the patient received prompt antimicrobial therapy, which contributed to the steady improvement in the patient's condition. Laboratory studies showed moderate coagulopathy with a median PT of 18.4 seconds (IQR = 16.8-20.0) and a median APTT of 42.6 seconds (IQR = 38.4-46.8). Haematological parameters showed moderate thrombocytopenia with a platelet count of 98 × 109/L (IQR = 86-110). The inflammatory profile demonstrated elevated CRP of 242 mg/L (IQR = 218-266), lower than vascular (324 mg/L), abdominal (286 mg/L), and thoracic cases (468 mg/L), and median procalcitonin of 18.2 ng/mL (IQR = 15.8-20.6), notably lower than other surgical subgroups.

The patient who underwent orthopaedic surgery had a favourable outcome after receiving intensive supportive care and early surgical debridement. This suggests that the elective nature of the procedure, with fewer baseline comorbidities, coupled with early detection and aggressive intervention, enabled timely management of the coagulopathic state. These findings emphasise the importance of remaining vigilant for signs of PF, especially in postoperative patients with early infection symptoms. Prompt recognition, immediate broad-spectrum antibiotics, and swift supportive care are critical to enhance survival in this patient population.

These findings carry significant clinical implications. The results emphasise the necessity for procedure-specific risk assessments and customised postoperative surveillance strategies. Early detection of systemic inflammatory responses, rapid initiation of appropriate antimicrobial therapy, and aggressive supportive care remain essential interventions across all surgical settings. The observed variation in the timing of onset and progression of AIPF across different surgical specialities highlights the need for targeted postoperative monitoring protocols. The notably poor outcomes seen in vascular surgery patients underscore the importance of enhanced surveillance and potentially more aggressive prophylactic measures in this high-risk group. Implementing tailored protocols could improve patient outcomes by addressing the unique challenges posed by each surgical speciality.

However, this analysis is not without limitations. The small sample size, especially in the thoracic and orthopaedic subgroups, restricts the generalizability of the findings. The retrospective design also precludes establishing definitive causal relationships between surgical factors and the development of AIPF, leaving room for potential bias and unmeasured confounding variables.

Future research should prioritise prospective studies involving larger, more diverse patient cohorts to confirm these observations and guide the development of evidence-based guidelines for the prevention and management of AIPF tailored to specific surgical disciplines. Further exploration into the interactions between surgical stress, immune response, and activation of the coagulation cascade could yield valuable insights into the pathophysiology of postoperative AIPF and inform targeted preventive strategies.

## Conclusions

AIPF is a rare but devastating postoperative complication with a high mortality rate. It occurs most commonly following abdominal and vascular surgeries and presents with sudden-onset widespread purpura, hypotension, and signs of multi-organ failure. Early recognition, aggressive supportive care, appropriate antimicrobial therapy, and timely surgical intervention are essential in improving patient outcomes. Understanding the interplay between surgical stress, immune response, and coagulation pathways remains crucial for advancing postoperative care and patient safety. Future multicentre, prospective studies are necessary to better understand risk factors and develop prevention strategies for this life-threatening condition. Given the high mortality associated with gram-negative infections, clinical protocols should consider incorporating routine gram-negative coverage in postoperative management for high-risk surgeries, with further research directed at refining these strategies to mitigate the devastating impacts of PF.
